# Monitoring activity-dependent bulk endocytosis with the genetically-encoded reporter VAMP4-pHluorin

**DOI:** 10.1016/j.jneumeth.2016.03.011

**Published:** 2016-06-15

**Authors:** Jessica C. Nicholson-Fish, Karen J. Smillie, Michael A. Cousin

**Affiliations:** Centre for Integrative Physiology, University of Edinburgh, Hugh Robson Building, George Square, Edinburgh EH8 9XD, Scotland, United Kingdom

**Keywords:** Endocytosis, Vesicle, Presynapse, VAMP4, Clathrin

## Abstract

**Background:**

Activity-dependent bulk endocytosis (ADBE) is the dominant mode of synaptic vesicle (SV) endocytosis during intense neuronal activity, implicating it as a major contributor to presynaptic plasticity under these stimulation conditions. However methods to monitor this endocytosis mode have been limited to either morphological or optical observation of the uptake of large fluid phase markers.

**New method:**

We present here a method to monitor ADBE using the genetically-encoded reporter VAMP4-pHluorin in primary neuronal cultures.

**Results:**

Individual nerve terminals expressing VAMP4-pHluorin display either an increase or decrease in fluorescence after stimulation terminates. The decrease in fluorescence reflects the slow acidification of large bulk endosomes to which VAMP4-pHluorin is selectively recruited. Use of VAMP4-pHluorin during sequential high frequency stimuli revealed that all nerve terminals perform ADBE, but not all do so in response to a single stimulus. VAMP4-pHluorin also displays a rapid activity-dependent decrease in fluorescence during high frequency stimulation, a response which is particularly prominent when expressed in hippocampal neurons. The molecular mechanism responsible for this decrease is still unclear, but is not due to loss of VAMP4-pHluorin from the nerve terminal.

**Comparison with existing methods:**

This method allows the selective reporting of ADBE for the first time, when compared to previous approaches using markers of fluid phase uptake.

**Conclusions:**

The development of VAMP4-pHluorin as a selective genetically-encoded reporter of ADBE increases the palette of approaches used to monitor this endocytosis mode both *in vitro* and *in vivo*.

## Highlights

• VAMP4-pHluorin is the first genetically-encoded activity-dependent bulk endocytosis reporter.
• It displays a complex fluorescent profile during high intensity stimulation.
• It reveals all nerve terminals can perform activity-dependent bulk endocytosis.

## Graphical abstract

**Figure:**
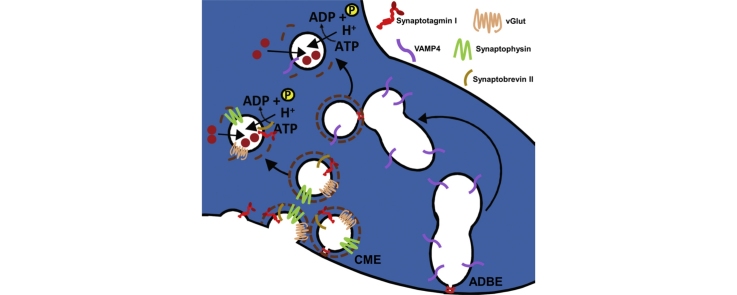

## 1 Introduction

Normal brain function requires that neurotransmission is sustained across a wide range of stimulation frequencies. The maintenance of neurotransmission is dependent on the efficient recycling of synaptic vesicle (SV) membrane and cargo after their insertion into the presynaptic plasma membrane during exocytosis (Saheki and De Camilli, 2012, Kokotos and Cousin, 2015). During sparse action potential simulation at physiological temperatures ultrafast endocytosis retrieves all SV membrane (Watanabe et al., 2013, Watanabe et al., 2014). When stimulation intensity is increased to trains of ≤10 Hz, the dominant endocytosis mode is clathrin-mediated endocytosis (CME) (Granseth et al., 2006, Zhu et al., 2009). CME has a fixed rate and limited capacity (Sankaranarayanan and Ryan, 2000), meaning that during high stimulus intensities SV membrane accumulates at the presynapse in the short term. In response to such stimuli, a different endocytosis mode is triggered called activity-dependent bulk endocytosis (ADBE). ADBE retrieves large areas of the presynaptic plasma membrane to form structures called bulk endosomes, from which new SVs are then generated (Clayton and Cousin, 2009a, Kokotos and Cousin, 2015). ADBE terminates immediately after the cessation of high frequency stimulation, meaning that it is tightly coupled to intense neuronal activity (Wu and Wu, 2007, Clayton et al., 2008). ADBE is also the dominant mode of membrane retrieval during these stimulation conditions (Clayton et al., 2008), implicating it in physiological events that rely on intense activity such as long-term potentiation or pathophysiological events such as epilepsy.

Considering the potential importance of ADBE to neuronal physiology there is an urgent requirement to have accurate assays that report both when and where this endocytosis mode is triggered in typical small nerve terminals of central neurons. Up to this point researchers have been limited to either morphological or optical approaches employing markers of the fluid phase. For morphology the fluid phase marker horse-radish peroxidase (HRP) is commonly used, since it can track the formation of both SVs *via* CME and bulk endosomes *via* ADBE using electron microscopy (Clayton et al., 2008, Clayton et al., 2009). Commonly employed optical approaches include either uptake of large fluorescent dextran (Holt et al., 2003, Clayton et al., 2008, Clayton et al., 2009) or uptake of styryl dyes such as FM1-43 and FM2-10 (Richards et al., 2000, Clayton et al., 2009). Large (typically 40 kDa) fluorescent dextrans such as tetramethylrhodamine-dextran (TMR-dextran) specifically report ADBE *via* size exclusion, since their large diameter hinders access to individual SVs with an internal diameter of 25 nm. The amphiphilic dye FM1-43 reports both CME and ADBE during intense stimulation, whereas under identical stimulation conditions FM2-10 only reports CME (Richards et al., 2000). The mechanism underlying this selective labelling may be due to the differences in their departition times from membranes inside the bulk endosome (Clayton and Cousin, 2008). These assays have provided key insights into the molecular mechanism of ADBE (Clayton and Cousin, 2009a, Kokotos and Cousin, 2015), however they all have limited time resolution, making dynamic measurements of this endocytosis mode difficult.

Genetically-encoded, pH-sensitive optical reporters have revolutionised the study of SV endocytosis in typical small central nerve terminals (Kavalali and Jorgensen, 2014). These reporters typically consist of an integral SV protein tagged at a lumenal domain with a pH-sensitive GFP moiety. However up to this point these reporters have been almost exclusively employed to monitor CME rather than ADBE, principally due to the stimulation intensities employed. An important reason that mild stimulation intensities were used to monitor CME was that it was assumed that the fluorescent output of these reporters would be confounded by the parallel trafficking of SV cargo *via* both CME and ADBE during high intensity stimulation. However the recent identification of an ADBE-specific cargo molecule—VAMP4 (Nicholson-Fish et al., 2015) now offers the opportunity to visualise this process in isolation from other endocytosis modes. We describe here methodologies to visualise ADBE in real time during physiological stimulation using the genetically-encoded reporter VAMP4-pHluorin.

## 2 Methods

### 2.1 Materials

Synaptophysin-pHluorin was provided by Prof. L. Lagnado (University of Sussex, UK). VAMP4-pHluorin was generated from an original sequence provided by Prof. E. Kavalali (UT Southwestern Medical Centre, Texas, USA) as described previously (Nicholson-Fish et al., 2015). DNA encoding the fluorescent protein mCerulean was from David Piston (Vanderbilt University, USA). Short hairpin RNA (shRNA) against clathrin heavy chain or a scrambled control inserted into vectors co-expressing mCerulean were described previously (Nicholson-Fish et al., 2015). Neurobasal media, B-27 supplement, penicillin/streptomycin, Minimal Essential Medium (MEM), and Lipofectamine 2000 were obtained from Thermofisher (Paisley, UK). All other reagents were obtained from Sigma–Aldrich (Poole, UK).

### 2.2 Tissue culture

All animal work was performed in accordance with the UK Animal (Scientific Procedures) Act 1986, under Project and Personal Licence authority and was approved by the Animal Welfare and Ethical Review Body at the University of Edinburgh. Specifically, all animals were killed by schedule 1 procedures in accordance with UK Home Office Guidelines.

Primary cultures of cerebellar neurons were prepared from the cerebella of 7 day old Sprague Dawley rat pups of both sexes. After removal, cerebella were run twice through a McIlwain tissue chopper (Mickle Laboratory Eng. Co., Ltd., Surrey, UK) using 375 μM intervals at 90° angles. The tissue was then placed in phosphate buffered saline (PBS, pH 7.4) supplemented with 14 mM glucose, 50 μM fatty acid free BSA, 3 mM MgSO_4_ and 0.25 mg/ml trypsin and incubated at 37° C for 20 min. After digestion with trypsin an equal volume of PBS supplemented with 8 μg/ml soybean trypsin inhibitor and 8 U/ml DNAse was added to the suspension and the sample was centrifuged at 1000 × *g* for 60 s. The supernatant was removed and the pellet was triturated using flame-polished glass Pasteur pipettes with bore sizes of decreasing diameters in a solution of PBS supplemented with 50 U/ml DNase, 50 μg/ml soybean trypsin inhibitor and 3 mM MgSO_4_. The cell suspension was then layered onto an Earle’s Balanced Salt Solution supplemented with 4% BSA and centrifuged at 3470 × *g* for 5 min. The pellet was resuspended in cerebellar culture medium (Minimal Essential Medium, 10% foetal bovine serum, 100 U/ml penicillin 100 μg/ml, 25 mM KCl, 33 mM glucose and 2 mM l-glutamine) and plated on 25 mm coverslips coated with poly-d-lysine in boric acid (100 mM, pH 8.5). After 24 h cerebellar culture media was replaced by an identical media solution supplemented with 10 μM cytosine β-d-arabinofuranoside to inhibit glial proliferation.

Dissociated primary hippocampal enriched neuronal cultures were prepared from E17.5 C57BL/6J mouse embryos of both sexes (obtained from an in-house breeding colony) by dissection of hippocampi into chilled PBS (pH 7.4). Hippocampi were digested in papain (0.3 U/ml) supplemented PBS at 37 °C for 20 min. Excess papain was removed and replaced with Supplemented DMEM/F12 (Dulbecco’s Modified Eagle Medium: Nutrient Mixture F-12) supplemented with 1% v/v penicillin/streptomycin solution and 10% w/v foetal bovine serum and triturated to obtain a single-cell suspension. The suspension was centrifuged for 5 min at 3470 × *g*. The supernatant was discarded and the pellet resuspended in Neurobasal medium supplemented with 2% B-27 supplement, 0.5 mM l-glutamine, and 1% v/v penicillin/streptomycin and then plated on 25 mm coverslips coated with poly-d-lysine in boric acid (100 mM, pH 8.5) with laminin spots. Cultures were maintained in the same Neurobasal media as above, however after 72 h culture the media was supplemented with media containing cytosine β-d-arabinofuranoside to a final concentration of 1 μM to inhibit glial proliferation.

Cerebellar neurons were plated at a density of 1.4 × 10^5^ cells, whereas hippocampal neurons were plated at a density of 5 × 10^4^ cells/coverslip. Cerebellar neurons were transfected between 5–7 days in culture whereas hippocampal neurons were transfected between 6–8 days in culture with Lipofectamine 2000 (Baker et al., 2015). Cerebellar neurons were imaged after 8–10 days in culture, whereas hippocampal neurons were imaged after 13–16 days.

### 2.3 Fluorescent live cell imaging

All experiments were performed with cultures enclosed inside a Warner Instruments (Hamden, USA) imaging chamber with embedded parallel platinum wires (RC-21BRFS) on the stage of a Zeiss (Oberkochen, Germany) Axio Observer A1 epifluorescence microscope. Cultures were visualised with a Zeiss Plan Apochromat x40 oil immersion objective (NA 1.3). Neurons were only chosen for analysis if they were located away from the stimulating electrodes to avoid potential fluorescence quenching artefacts. Time-lapse images were captured using a Zeiss AxioCam MRm Rev.3 digital camera at 4 s intervals. All images were processed offline using Image J 1.43 software (National Institute of Health, USA). Time-lapse videos were processed using the ImageJ plugin Time Series Analyser (http://rsb.info.nih.gov/ij/plugins/time-series.html). Identically sized regions of interest were placed over nerve terminals and the total fluorescence recorded over time. All statistical analyses were performed using Microsoft Excel and GraphPad Prism software.

In all experiments cells were subject to continuous perfusion with imaging buffers specific for each neuronal culture type. Prior to imaging, cerebellar neurons were repolarized for 10 min in imaging buffer consisting of (170 mM NaCl, 3.5 mM KCl, 400 μM KH_2_PO_4_, 20 mM TES [*N*-tris[hydroxy-methyl]-methyl-2-aminoethane-sulfonic acid], 5 mM NaHCO_3_, 5 mM glucose, 1.2 mM Na_2_SO_4_, 1.2 mM MgCl_2_, 1.3 mM CaCl_2_ at pH 7.4). This imaging buffer was then used in all subsequent steps. For experiments with hippocampal cultures the repolarization step was omitted and neurons were continuously bathed in a modified imaging buffer (136 mM NaCl, 2.5 mM KCl, 2 mM CaCl_2_, 1.3 mM MgCl_2_, 10 mM glucose, 10 mM HEPES, 10 μM CNQX, 50 μM AP-5 at pH 7.4). All experiments were performed at room temperature.

#### 2.3.1 Fluorescent imaging of synaptophysin-pHluorin

Synaptophysin-pHluorin (syp-pHluorin) fluorescence in transfected neurons was visualised using 500 nm excitation and a long pass (>535 nm) emission filter. A baseline of fluorescence was acquired for one minute (15 frames) prior to stimulation with a train of action potentials and further image acquisition for at least 3 min after. At the end of each experiment cultures were challenged with alkaline imaging buffer to reveal total pHluorin fluorescence (50 mM NH_4_Cl substituted for 50 mM NaCl for both cerebellar and hippocampal imaging buffers). Circular regions of interest of diameter 5 × 5 pixels were employed (approx. 20 square pixels). Only regions that displayed an increase in fluorescence in response to action potential stimulation were selected for analysis. The average trace from each experiment was normalised to baseline (Δ*F*/*F*_0_) and then to peak height. When required, traces were corrected for photo-bleaching by subtracting a mono-exponential decay curve fitted to the syp-pHluorin signal before stimulation. In all cases *n* refers to the number of individual coverslips examined.

#### 2.3.2 Fluorescent imaging of VAMP4-pHluorin

**Fig. 1:**
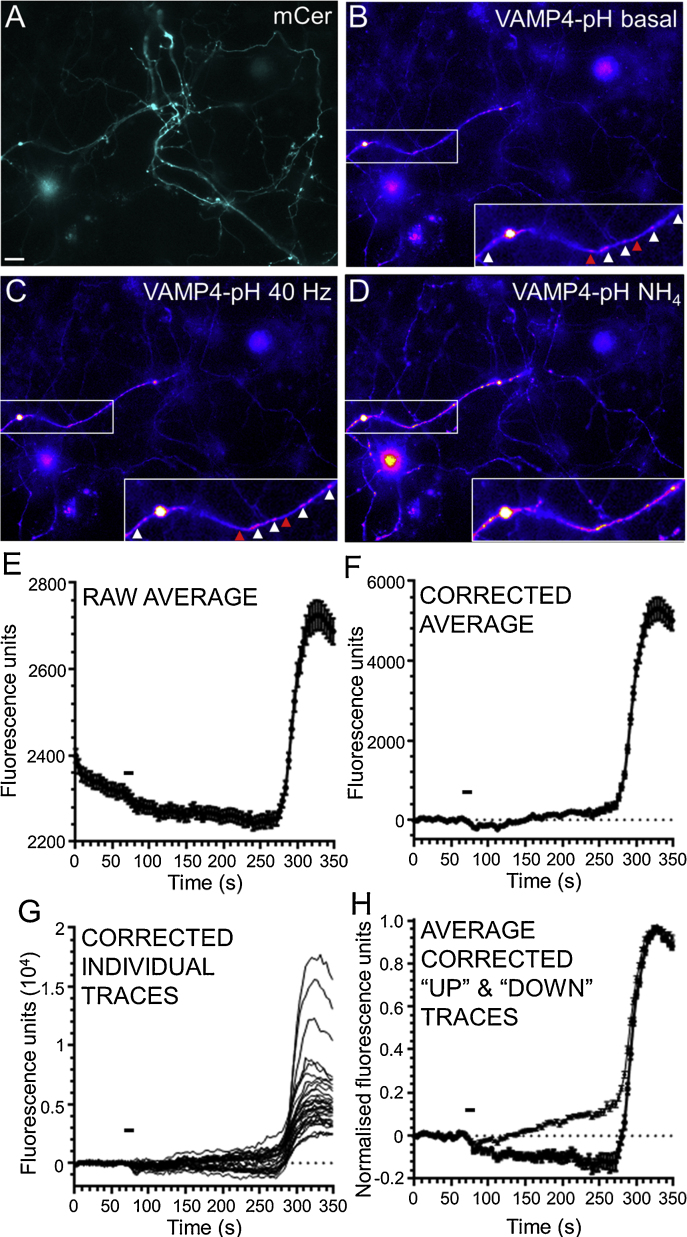
Protocol for analysis of VAMP4-pHluorin traces. (A–D) Representative images display hippocampal neurons that were transfected with both VAMP4-pHluorin (VAMP4-pH) and mCerulean empty vector (mCer). Neurons were initially located using mCerulean fluorescence and then the fluorescent response from VAMP4-pHluorin was monitored over time in response to a train of 400 action potentials (40 Hz). Images display the initial mCer image (A), then VAMP4-pH images either before (Basal, B), 2 min after stimulation (40 Hz, C) or after exposure to alkaline buffer (NH_4_, D). Scale bar represents 10 μm. Magnified insets display alterations in VAMP4-pH signal with white arrowheads indicating “Up” responses, and red arrowheads indicating “Down” responses. (E) Representative average time course of VAMP4-pHluorin fluorescence in response to action potential stimulation as indicated by bar (raw average). (F) The same trace after decay correction with a non-linear regression curve fitted to the baseline (corrected average). After correction individual decay corrected traces (G) were then separated into two subsets: those which had a relative decrease in fluorescence 2 min after stimulation and those which had a relative increase (H, average corrected “Up” and “Down” traces). In all cases bar represents period of stimulation, error bars represent ±SEM (*n* = 33 nerve terminals from a single representative experiment). (For interpretation of the references to colour in this figure legend, the reader is referred to the web version of this article.)

Experiments monitoring VAMP4-pHluorin fluorescence were performed in an identical manner to those with syp-pHluorin with the following exceptions. VAMP4-pHluorin was always co-transfected with a mCerulean vector to allow identification of transfected neurons (visualised at 430 nm excitation, >535 nm emission, Fig. 1A). Neurons were selected for analysis based on their morphology, specifically if they displayed no signs of ill health, such as blebbing or crumbling of neurites. Visualisation of VAMP4-pHluorin at 500 nm excitation (emission > 535 nm) ensured spectral separation from mCerulean fluorescence (Fig. 1B–D). During the image acquisition process 3 × 3 pixel binning was performed in either Axiovision or Zen Pro software (both Zeiss Ltd.) due to the low emitted fluorescence of VAMP4-pHluorin transfected neurons. Regions of interest of diameter 3 × 3 binned pixels were employed (equivalent to 9 × 9 unbinned pixels, 63 square pixels). VAMP4-pHluorin undergoes significant photobleaching over time, therefore a longer baseline period was acquired, with a non-linear regression curve then fitted to the first 19 frames (76 s) of baseline for each nerve terminal trace in GraphPad Prism. The equation derived from these regression curves was then subtracted from the original data (compare Fig. 1E and F).

In addition to analysis of the VAMP4-pHluorin average response from multiple nerve terminals in the same field of view, further analysis was performed on the individual nerve terminal responses. Nerve terminals were initially screened either on their immediate response to stimulation (for hippocampal neurons) or whether they had deviated from baseline immediately prior to exposure to alkaline imaging buffer (for cerebellar neurons). Screened responses from individual nerve terminals (Fig. 1G) were normalised to the maximum fluorescence value in the presence of alkaline imaging buffer with “1” representing maximal VAMP4-pHluorin fluorescence in the presence of alkaline buffer and “0” representing baseline. After this step, responses were segregated on the following basis: individual nerve terminals which displayed an increase in fluorescence above baseline 2 min after stimulation were classified as “Up”, whereas those which displayed a decrease were classified as “Down” (Fig. 1H). Responses were segregated in Microsoft Excel using the formula below.

“IF(‘Normalised  Traces’!BH5 < AVERAGE(‘Normalised  Traces’!B5:O5),▼,▲)”

where BH5 = the fluorescent value at 2 min and AVERAGE is the average decay-corrected fluorescent baseline.

In specific instances two sequential stimulations were performed that were separated by 10 min. The protocol for this experiment was identical to that described above, apart from the fact that individual nerve terminal responses were segregated into four populations, being (1) “S1 ‘Up’, S2 “Up”; (2) “S1 ‘Up’, S2 “Down”; (3) “S1 ‘Down’, S2 “Up” and (4) “S1 ‘Down’, S2 “Down”. In all cases *n* refers to the number of individual coverslips examined, unless stated otherwise.

## 3 Results and discussion

Exquisitely pH-sensitive GFP moieties (pHluorins) that are fused to the lumenal domain of a SV cargo protein are widely used as optical reporters of SV recycling in central neurons. The fluorescence of pHluorin is quenched within the acidic lumen of the SV and it is unquenched at neutral pH. This therefore permits visualisation of the dynamic, activity-dependent trafficking of this reporter during both exocytosis and endocytosis (Kavalali and Jorgensen, 2014). The extent and speed of SV exocytosis can be estimated by monitoring the increase in fluorescence of the reporter during stimulation as it transitions from an acidic to a neutral extracellular environment.

**Fig. 2:**
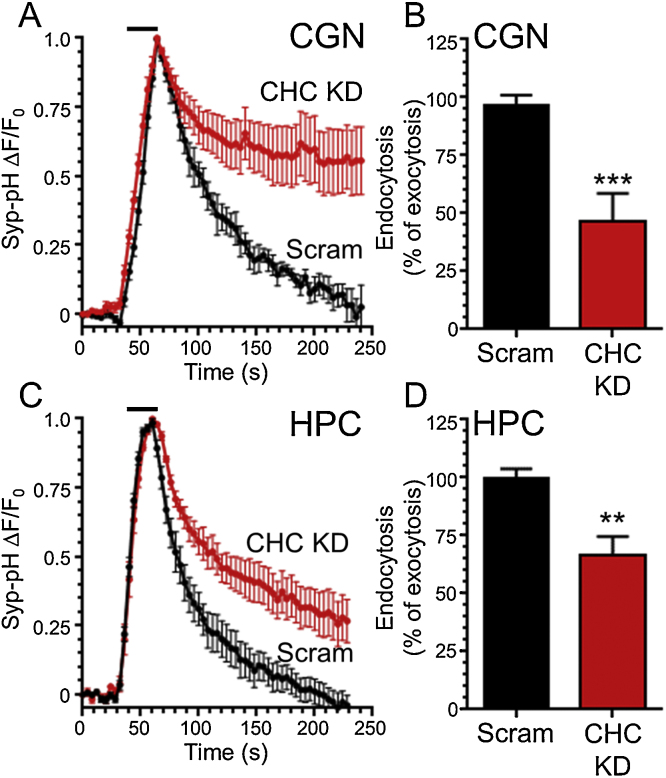
Knockdown of clathrin heavy chain inhibits synaptophysin-pHluorin retrieval during low frequency stimulation. Hippocampal (HPC) or cerebellar (CGN) neurons that were transfected with synaptophysin-pHluorin (syp-pHluorin) and either shRNA against clathrin heavy chain (CHC KD) or a scrambled version (Scram). Neurons were stimulated with a train of 300 action potentials (10 Hz) as indicated by bar. (A, C) The average time course of syp-pHluorin fluorescence in response to action potential stimulation is displayed for either CGN (A) or HPC (C) neurones; *F*/*F*_0_ ± SEM. (B, D) The average extent of syp-pHluorin retrieval (endocytosis) at 200 s is displayed measured as a percentage of exocytosis (peak height) for either CGN (B) or HPC (D) neurons. (CGN; Scram shRNA *n* = 5, CHC KD *n* = 8; HPC; Scram shRNA *n* = 5, CHC KD *n* = 5; ***p < 0.001, **p < 0.01, Student’s *t* test).

Standard protocols for both stimulation of neurons expressing pHluorin reporters and subsequent monitoring of their traffic are widely available and employed (Kavalali and Jorgensen, 2014). An example is shown here for syp-pHluorin in Fig. 2. Cerebellar or hippocampal neurons expressing syp-pHluorin were stimulated with a train of action potentials (10 Hz, 30 s) and then allowed to recover for 3 min. This protocol was chosen since minimal triggering of ADBE is observed at frequencies ≤10 Hz (Clayton et al., 2008, Wenzel et al., 2012). During neuronal activity syp-pHluorin displays a rapid increase in fluorescence followed by a slow post-stimulation decrease (Fig. 2). Since this relatively mild stimulation protocol does not trigger ADBE (Clayton et al., 2008), the kinetics of CME can be estimated by determining the time constant of this post-stimulation fluorescence decrease (Fig. 2). This estimation can be performed since CME is rate limiting compared to subsequent SV acidification, with the latter occurring within 3–5 s (Atluri and Ryan, 2006, Granseth et al., 2006) (but see Egashira et al., 2015). To confirm that CME was the dominant endocytosis mode during this stimulation protocol, clathrin heavy chain (CHC) was depleted from both cerebellar and hippocampal neurons using shRNA. Knockdown in both neuronal cell types was extensive (cerebellar neurones: CHC shRNA 22.8 ± 7.0 of untransfected control, scrambled shRNA 105.2 ± 4.5; hippocampal neurones: CHC shRNA 19.1 ± 1.2 of untransfected, scrambled shRNA 103.8 ± 5.4 (Nicholson-Fish et al., 2015)). CHC depletion greatly retarded the retrieval of syp-pHluorin in both neuronal types when compared to a scrambled control shRNA (Fig. 2). Thus CME is the dominant mode of SV endocytosis during low frequency stimulation at room temperature (Granseth et al., 2006, Zhu et al., 2009).

### 3.1 Synaptophysin-pHluorin does not report ADBE

**Fig. 3:**
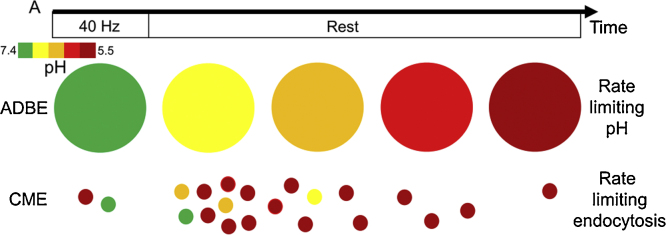
CME and ADBE have different rate-limiting steps. Schematic displays the time course of both CME and ADBE during high frequency stimulation. During stimulation bulk endosomes are formed by ADBE which are neutral in pH (represented by green, as indicated by pH key). CME only forms a few SVs during stimulation which are quickly acidified (represented by red). After stimulation no more bulk endosomes are formed by ADBE. The endosomes that were formed during ADBE slowly acidify over time (represented by the transition from green to red). Most SVs formed by CME occur after stimulation with the number decreasing as a function of time. All are rapidly acidified. Therefore bulk endosome acidification is rate limiting for ADBE, whereas SV retrieval is rate limiting for CME. (For interpretation of the references to colour in this figure legend, the reader is referred to the web version of this article.)

When stimulation intensity increases, the interpretation of pHluorin responses becomes more complex. This is because ADBE is triggered in addition to CME, with ADBE being the dominant endocytosis mode during these stimulation conditions (Clayton et al., 2008). ADBE is tightly coupled to intense activity and only occurs concurrent with action potential stimulation. During high intensity stimulation ADBE generates endosomes direct from the plasma membrane that have an average diameter of approximately 150 nm (Kokotos and Cousin, 2015). This means that these endosomes have an internal volume that is approximately 50 fold larger than a 40 nm SV (using volume = 4/3 × *π* × radius^3^). Therefore the pHluorin response that occurs after high intensity stimulation should predominantly be a function of bulk endosome acidification, since these endosomes have already formed when stimulation terminates. This is particularly true when one considers that the V-type ATPases that are responsible for acidifying both SVs and endosomes are highly limited at the presynapse (Takamori et al., 2006, Wilhelm et al., 2014). These studies have revealed that there are between 1–2 copies of these ATPases on SVs and that within the nerve terminal, SVs contain >75% of all of these transporter molecules (Wilhelm et al., 2014). This means that there is a very limited pool of excess V-ATPase molecules that bulk endosomes can draw from to accelerate acidification. One would therefore predict that commonly used pHluorins could not report either CME or ADBE accurately either during or after high frequency stimulation due to the confounding and opposite effects of SV/endosome formation and their subsequent acidification (Fig. 3).

We have shown that this hypothesis was not supported by experiments performed in both cerebellar and hippocampal cultures challenged with trains of high frequency action potentials. Firstly the fluorescence of syp-pHluorin could be fully quenched by application of an impermeant weak acid immediately after stimulation, suggesting it was not accumulated into slowly acidifying bulk endosomes (Nicholson-Fish et al., 2015). Second, a series of pharmacological and molecular interventions that inhibited ADBE had no effect on the evoked responses of commonly used pHluorin reporters such as syp-pHluorin, synaptotagmin-1-pHluorin, synaptobrevin II-pHluorin and vGLUT1-pHluorin (Nicholson-Fish et al., 2015). Finally, either a small molecule inhibitor of CME or genetic knockdown of CHC strongly inhibited the post-stimulation fluorescence decrease of either syp-pHluorin or synaptotagmin-1-pHluorin (Nicholson-Fish et al., 2015). Thus commonly used pHluorins are preferentially retrieved by CME during intense stimulation, even though ADBE is the dominant endocytosis mode under these conditions.

**Fig. 4:**
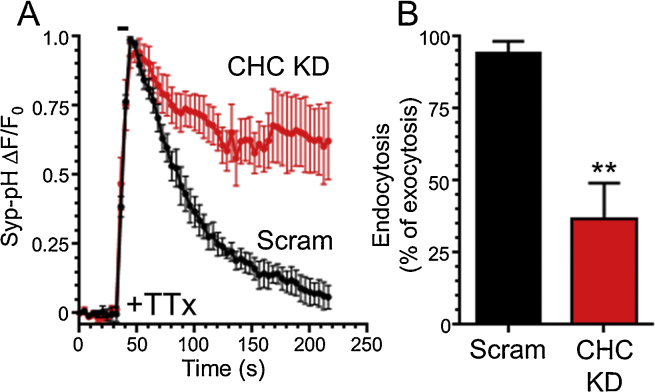
The clathrin-dependence of synaptophysin-pHluorin retrieval is not affected by chronic silencing of spontaneous network activity. (A) Hippocampal neurons were co-transfected with synaptophysin-pHluorin (syp-pHluorin) and either shRNA against clathrin heavy chain (CHC KD) or a scrambled version (Scram). In all cases culture medium was supplemented with 100 nM tetrodotoxin (TTx) for 48 h prior to the experiment. Neurons were then transferred to hippocampal imaging buffer and stimulated with a train of 400 action potentials (40 Hz). The evoked average fluorescent response of syp-pHluorin is displayed Δ*F*/*F*_0_ ± SEM with stimulation indicated by bar. (B) The average extent of syp-pHluorin retrieval (endocytosis) at 200 s is displayed measured as a percentage of exocytosis (peak height) ± SEM (Scram *n* = 5, CHC KD *n* = 4, **p < 0.01, Student’s *t* test).

In the study above, CHC was depleted using shRNA delivered by transient transfection, whereas a separate study using lentiviral delivery of shRNA against CHC had no effect on synaptotagmin-1-pHluorin retrieval during high frequency stimulation (Kononenko et al., 2014). It is possible that the disparity between the two studies results from the selective transduction of excitatory neurons by lentivirus (Nathanson et al., 2009). In this scenario, long-term depletion of CHC in excitatory neurons may potentially “silence” these cultures, leading to an adaptive response resulting in SV cargo retrieval occurring *via* a clathrin-independent endocytosis mode. To test this, we incubated hippocampal cultures that had been transiently transfected with CHC shRNA (or a scrambled version) with the sodium channel antagonist tetrodotoxin for 2 days. This manoeuvre silences spontaneous activity within the culture and thus mimics the potential loss of excitatory input. These cultures were then challenged with a train of high frequency action potentials (40 Hz 10 s) to determine whether these neurons adapted to silencing by altering the mode of SV endocytosis. In neurons expressing scrambled CHC shRNA we observed a typical syp-pHluorin response with a fast initial increase and a slow decrease, indicating endocytosis and SV acidification (Fig. 4). However in neurons expressing CHC shRNA, the syp-pHluorin response did not recover to baseline, indicating an arrest of syp-pHluorin retrieval *via* CME (Fig. 4). Therefore even after chronic silencing of neuronal activity CME still retrieves SV cargo during high frequency stimulation, even though ADBE is the dominant endocytosis mode under these conditions.

### 3.2 VAMP4-pHluorin reports ADBE

**Fig. 5:**
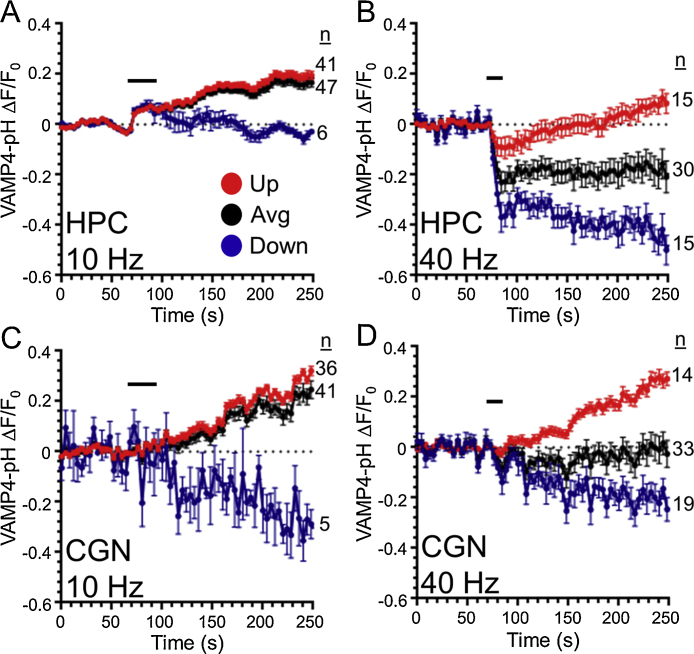
Frequency dependency of the VAMP4-pHluorin response. (A) Hippocampal (HPC, A, B) or cerebellar (CGN, C, D) neurons were transfected with both VAMP4-pHluorin and mCerulean and then stimulated with a train of either 400 action potentials (40 Hz, B, D) or 300 action potentials (10 Hz, A, C) indicated by bars respectively. Black traces display the average (Avg) VAMP4-pHluorin response from a single experiment, whereas red and blue traces represent the average “Up” and “Down” traces from that experiment respectively. All traces are displayed ±SEM with very few nerve terminals displaying a “Down” profile after 10 Hz stimulation (HPC 10 Hz; *n* = 47 nerve terminals total, *n* = 41 Up, *n* = 6 Down; 40 Hz; *n* = 30 total, *n* = 15 Up, *n* = 15 down; CGN 10 Hz; *n* = 41 total, *n* = 36 Up, *n* = 5 Down; CGN 40 Hz; *n* = 33 total, *n* = 14 Up, *n* = 19 Down). (For interpretation of the references to colour in this figure legend, the reader is referred to the web version of this article.)

In contrast to the commonly employed pHluorin reporters described above, VAMP4-pHluorin faithfully reports ADBE and displays an atypical response on challenge with a train of high frequency action potentials (Raingo et al., 2012, Nicholson-Fish et al., 2015). When expressed in hippocampal neurons the average nerve terminal response is a dramatic initial drop in fluorescence, followed by a slow recovery back to baseline (Fig. 5B). In contrast when the VAMP4-pHluorin response is examined in cerebellar neurons the average fluorescent response is the almost negligible (Fig. 5D). However a more complex picture emerges when the responses of individual nerve terminals are investigated. In the period after stimulation there is a sharp divergence in the behaviour of individual nerve terminals in their VAMP4-pHluorin response. In both cerebellar and hippocampal neurons, some nerve terminals display a slow increase in fluorescence (Fig. 5B and D). This slow increase is proposed to be asynchronous release, which is dependent on the copy number of VAMP4 on SVs. Asynchronous release is inhibited in the absence of VAMP4, whereas VAMP4 overexpression increases the extent of this form of release (Raingo et al., 2012). The prolonged increase in fluorescence over the course of minutes in these nerve terminals suggests that the later component of the response cannot be solely attributed to asynchronous release. However it is clear that it does reflect the sustained fusion of VAMP4-pHluorin expressing SVs minutes after stimulation terminates. The physiological role of this sustained release will be a key question for future investigation. Other nerve terminals display the opposite behaviour, with a continued slow downwards decrease in VAMP4-pHluorin fluorescence (Fig. 5B and D). These nerve terminals can be categorised by segregating them on the basis of whether their fluorescence levels were either higher or lower than their initial starting baseline 2 min after termination of stimulation. We categorised nerve terminals with an increased post-stimulation fluorescence (equating to the occurrence of asynchronous release) as “Up” whereas those with lower post-stimulation fluorescence as “Down” (Fig. 5).

### 3.3 Slow post-stimulation decrease in VAMP4-pHluorin fluorescence reports bulk endosome acidification

The “Down” responses are nerve terminals undergoing ADBE, and are a result of bulk endosomes that were formed during intense stimulation subsequently acidifying in this post-stimulation period. We state this since the number of “Down” responses was greatly reduced by either pharmacological or molecular interventions that arrested ADBE (Nicholson-Fish et al., 2015). Furthermore, nerve terminals displaying “Down” responses are almost completely absent when either cerebellar or hippocampal cultures were stimulated at intensities that did not trigger ADBE (Nicholson-Fish et al., 2015) (Fig. 5A and C). As a final proof, either cerebellar or hippocampal cultures were challenged with an impermeant acid solution after high intensity stimulation. This determined whether VAMP4-pHluorin fluorescence was inaccessible to quenching and thus not on the plasma membrane. In both culture systems the acid solution could not quench the signal post-stimulation, indicating that VAMP4-pHluorin was within a neutral compartment within the nerve terminal (Nicholson-Fish et al., 2015). This protection was not observed after 10 Hz stimulation (which does not trigger ADBE (Clayton et al., 2008)), indicating all fluorescent VAMP4-pHluorin was on the plasma membrane after this stimulation protocol (Nicholson-Fish et al., 2015). This confirmed that VAMP4-pHluorin is inside a slowly acidifying compartment such as a bulk endosome only after high frequency stimulation.

One of the results revealed by using VAMP4-pHluorin was that not all nerve terminals performed ADBE on challenge with intense stimulation. This agreed with previous studies in cerebellar neurones using uptake of the fluorescent fluid phase marker TMR-dextran to monitor the proportion of nerve terminals undergoing ADBE. In that study approximately 35% of cerebellar nerve terminals accumulated TMR-dextran during intense stimulation (Clayton and Cousin, 2009b) compared to 65% observed with VAMP4-pHluorin (Nicholson-Fish et al., 2015). This indicates that VAMP4-pHluorin is a more sensitive measure of ADBE than fluid phase uptake, since the non-directed accumulation of TMR-dextran may not efficiently capture all nerve terminals undergoing ADBE. In agreement approximately 20% of hippocampal nerve terminals were reported to undergo ADBE when monitored using TMR-dextran (Wenzel et al., 2012) but approximately 40% did so when measured using VAMP4-pHluorin (Nicholson-Fish et al., 2015).

### 3.4 All nerve terminals have the ability to perform ADBE

**Fig. 6:**
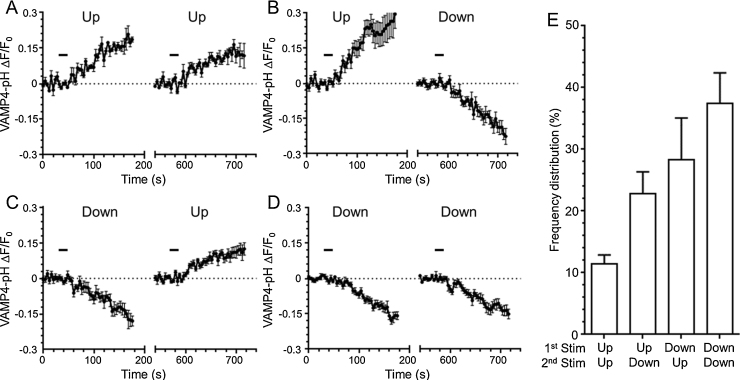
Nerve terminals can undergo ADBE, but not all within the same stimulus train. Cerebellar neurons were transfected with both VAMP4-pHluorin and mCerulean and then challenged with 2 trains of 400 action potentials (40 Hz) 10 min apart (indicated by bar). Responses were pooled into those nerve terminals that displayed “Up” behaviour on both stimuli (A), first “Up” then “Down” (B), first “Down” then “Up” (C) or both “Down” (D), all Δ*F*/*F*_0_ ± SEM. (E) Quantification of the proportions of the four response profiles in nerve terminals ±SEM (*n* = 6).

These results suggest that there may be specific nerve terminals that cannot perform ADBE. This hypothesis can be tested directly using the VAMP4-pHluorin assay. To determine this, we delivered two sequential high frequency stimuli separated by a 10 min interval and compared the evoked response to these two stimuli in the same nerve terminals. We grouped the nerve terminal populations that either displayed ADBE in either both trains of stimuli (Down/Down), neither stimuli (Up/Up) or one of the stimuli (Up/Down, Down/Up) (Fig. 6A–D). When the proportion of nerve terminals that displayed each of these four behaviours was calculated, we found that approximately 65% performed ADBE at either the first or second stimulation (Fig. 6E). Intriguingly, it was not the same population of nerve terminals that performed ADBE on each occasion. When the number of nerve terminals that performed ADBE at least once across both high frequency stimuli was calculated, it revealed that approximately 90% fell in this cohort (Fig. 6E). Thus under these conditions almost all nerve terminals possess the ability to perform ADBE, however this endocytosis mode is not uniformly triggered during intense stimulation. The reasons for this are currently under investigation, but may originate from either the prior stimulation experienced, the current SV pool size or input from post-synaptic partners. Regardless this is an important observation, since it means that VAMP4-positive SVs can be generated in almost all nerve terminals to mediate asynchronous release, even though a specific nerve terminal may not undergo ADBE during a specific stimulus train.

It is entirely possible that a nerve terminal may undergo both ADBE and asynchronous release in response to the same high frequency stimulus. In this case the VAMP4-pHluorin response potentially reflects the balance of these two separate processes. It is likely that ADBE dominates the VAMP4-pHluorin response in this scenario, since when this endocytosis mode is inhibited almost all nerve terminals displayed an “Up” response (Nicholson-Fish et al., 2015). Thus it seems likely that most nerve terminals undergo asynchronous release during intense stimulation, however this is only reported by VAMP4-pHluorin in nerve terminals that are undergoing either little or no ADBE during that specific stimulus.

### 3.5 Activity-dependent VAMP4-pHluorin trafficking during intense stimulation

**Fig. 7:**
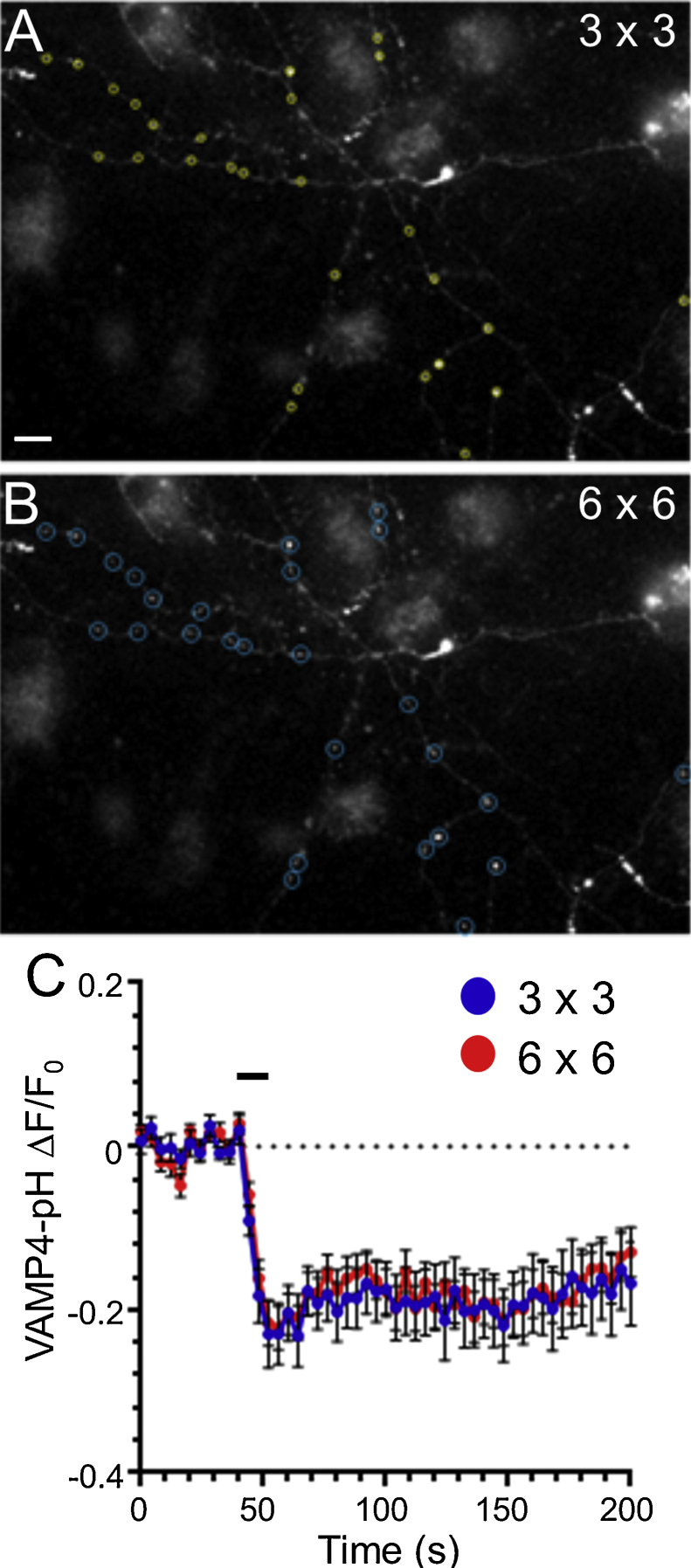
VAMP4-pHluorin does not escape the nerve terminal during intense stimulation. (A, B) Representative images showing a hippocampal neuron transfected with VAMP4-pHluorin with regions of interest of either 3 × 3 (A) or 6 × 6 (B) pixels. Scale bar equivalent to 10 μm. (C) Time course of the VAMP4-pHluorin response from a single representative experiment either at the nerve terminal (3 × 3) or including adjacent axonal regions (6 × 6) Δ*F*/*F*_0_ ± SEM (*n* = 40 nerve terminals). The average trace from the 6 × 6 pixel area was identical to that of a smaller region of interest, indicating that the immediate activity-dependent downstroke was due to internalisation of VAMP4-pHluorin and not its escape from the nerve terminal.

The slow “Down” VAMP4-pHluorin response reflects the acidification of the bulk endosome after its prior generation during high frequency stimulation. However there is another component to the response that is particularly prevalent in hippocampal neurons. This is a fast, activity-dependent drop in fluorescence during the stimulation period itself. The most obvious explanation for this rapid drop is that it is due to diffusion of VAMP4-pHluorin from the nerve terminal after SV fusion. However this is not the case, since doubling the size of the acquisition area (to include neighbouring axonal regions) has no effect on the extent or speed of this drop (Fig. 7).

The most parsimonious explanation for this activity-dependent decrease in fluorescence is that there is an immediate internalisation of VAMP4-pHluorin into an endocytic compartment which then rapidly acidifies. However this is unlikely to be a bulk endosome, since these compartments acidify much more slowly than SVs. Another possibility is that VAMP4-pHluorin is retrieved and the endocytic carrier immediately fuses with an acidified endosome (possibly a bulk endosome formed by a previous train of stimuli). Therefore several questions remain regarding this event, including (1) why is this event more prominent in hippocampal neurons when compared to cerebellar neurons, (2) what is the underlying mechanism and critically, (3) is it related to, or essential for, ADBE? While we do not have an answer for the first question, we do have information regarding the other two.

In terms of the underlying mechanism, the activity-dependent internalisation of VAMP4-pHluorin is dependent on its interaction with adaptor proteins. This is because mutant VAMP4-pHluorin that has a key dileucine motif ablated (L25A) does not display this activity-dependent retrieval when expressed in either hippocampal or cerebellar neurons (Nicholson-Fish et al., 2015). ADBE is arrested by depletion of VAMP4 using shRNA (monitored using TMR-dextran uptake). Interestingly the same L25A mutant cannot rescue ADBE in this knockdown system, suggesting that this initial event may be required for progression of ADBE (Nicholson-Fish et al., 2015). Adaptor-protein complexes are usually required to cluster cargo during clathrin-mediated endocytosis events (Kelly and Owen, 2011, Saheki and De Camilli, 2012). It is notable that either knockdown of CHC or application of the clathrin inhibitor pitstop-2 arrest this activity-dependent drop, suggesting that it may also be a clathrin-dependent event (Nicholson-Fish et al., 2015). However the kinetics of this drop are extremely rapid, and do not correlate with current estimates of the speed of either CME or SV acidification (Atluri and Ryan, 2006, Granseth et al., 2006). Furthermore the fact that the dileucine motif of VAMP4 specifically interacts with AP-1 and not AP-2 (Peden et al., 2001, Hinners et al., 2003) suggests that a canonical CME pathway is not responsible for this retrieval event.

A key remaining question is whether this activity-dependent retrieval is an obligatory step in ADBE. In support, adaptor-binding deficient VAMP4 cannot support ADBE (Nicholson-Fish et al., 2015). Furthermore this activity-dependent decrease is not observed in either hippocampal or cerebellar neurons at stimulation intensities that do not trigger ADBE (10 Hz, Fig. 5A and C). An important argument against an obligatory requirement in ADBE is the fact that inhibition of CME abolishes the initial activity-dependent drop but not the subsequent post-simulation “Down” response in either hippocampal or cerebellar neurons (Nicholson-Fish et al., 2015). This therefore suggests that an AP-1-dependent, clathrin-independent retrieval process retrieves VAMP4 as a first, essential step of ADBE. It will be of critical importance to determine the underlying mechanism of VAMP4 retrieval in this context.

### 3.6 Future use

The study of ABDE has been hindered by a lack of reliable tools with which to monitor its triggering and progression in central neurons. We describe here a protocol for use of the first genetically-encoded reporter of ADBE to visualise this key endocytosis mode in typical small central nerve terminals. The identification of VAMP4-pHluorin as a specific ADBE cargo molecule opens up potential for the delivery of different “payloads” specifically to bulk endosomes to manipulate their function. For example, different genetically encoded reporters/enzymes/chelators could be fused to the VAMP4 luminal domain to specially track ADBE derived SVs or manipulate their generation. Similarly, this system could also be used to deliver small molecules to the bulk endosome by fusing acceptors to VAMP4. These experiments could reveal important molecular information regarding the ADBE pathway after bulk endosome formation.

VAMP4-pHluorin has important advantages over previously employed optical markers of ADBE such as TMR-dextran. TMR-dextran simply labels the fluid phase and is selectively accumulated by ADBE due to size exclusion from CME. However it cannot be donated to SVs that are generated from bulk endosomes (Clayton and Cousin, 2009b) making it unsuitable for the study of multiple cycles of ADBE. In contrast VAMP4-pHluorin allows multiple cycles of ADBE to be visualised in the same nerve terminal. This will permit studies that examine presynaptic adaptation to high frequency input and also whether specific patterns of lower intensity stimulation can trigger ADBE during more prolonged firing. The characterisation of the VAMP4-pHluorin protocol also presents the exciting opportunity to simultaneously monitor two major endocytosis modes (CME and ADBE) in the same nerve terminal by exploiting red-shifted variants of GFP such as mOrange2 fused to common SV cargoes (Raingo et al., 2012, Ramirez et al., 2012). This will facilitate studies of their interplay at the presynapse in response to specific patterns of physiological stimuli and/or molecular interventions in primary cultures derived from both wild-type and mutant mice.

Finally, the discovery of VAMP4-pHluorin as a selective reporter of ADBE opens up the possibility of incorporating the probe at the genomic level to facilitate *in vivo* studies of this endocytosis mode. This approach has yielded important information in previous studies in both synaptobrevin II-pHluorin-expressing invertebrate and mammalian systems (Araki et al., 2005, Li et al., 2005, Dason et al., 2010, Linares-Clemente et al., 2015). The potential power of this approach is still untapped, however it may offer the tantalising possibility of monitoring ADBE in intact neuronal circuits, in real time, *in vivo*.

## References

[bib0005] Araki R., Sakagami H., Yanagawa Y., Hikima T., Ishizuka T., Yawo H. (2005). Transgenic mouse lines expressing synaptopHluorin in hippocampus and cerebellar cortex. Genesis.

[bib0010] Atluri P.P., Ryan T.A. (2006). The kinetics of synaptic vesicle reacidification at hippocampal nerve terminals. J. Neurosci..

[bib0015] Baker K., Gordon S.L., Grozeva D., van Kogelenberg M., Roberts N.Y., Pike M., Blair E., Hurles M.E., Chong W.K., Baldeweg T., Kurian M.A., Boyd S.G., Cousin M.A., Raymond F.L. (2015). Identification of a human synaptotagmin-1 mutation that perturbs synaptic vesicle cycling. J. Clin. Investig..

[bib0020] Clayton E.L., Cousin M.A. (2008). Differential labelling of bulk endocytosis in nerve terminals by FM dyes. Neurochem. Int..

[bib0025] Clayton E.L., Cousin M.A. (2009). The molecular physiology of activity-dependent bulk endocytosis of synaptic vesicles. J. Neurochem..

[bib0030] Clayton E.L., Cousin M.A. (2009). Quantitative monitoring of activity-dependent bulk endocytosis of synaptic vesicle membrane by fluorescent dextran imaging. J. Neurosci. Methods.

[bib0035] Clayton E.L., Evans G.J., Cousin M.A. (2008). Bulk synaptic vesicle endocytosis is rapidly triggered during strong stimulation. J. Neurosci..

[bib0040] Clayton E.L., Anggono V., Smillie K.J., Chau N., Robinson P.J., Cousin M.A. (2009). The phospho-dependent dynamin–syndapin interaction triggers activity-dependent bulk endocytosis of synaptic vesicles. J. Neurosci..

[bib0045] Dason J.S., Smith A.J., Marin L., Charlton M.P. (2010). Vesicular sterols are essential for synaptic vesicle cycling. J. Neurosci..

[bib0050] Egashira Y., Takase M., Takamori S. (2015). Monitoring of vacuolar-type H+ ATPase-mediated proton influx into synaptic vesicles. J. Neurosci..

[bib0055] Granseth B., Odermatt B., Royle S.J., Lagnado L. (2006). Clathrin-mediated endocytosis is the dominant mechanism of vesicle retrieval at hippocampal synapses. Neuron.

[bib0060] Hinners I., Wendler F., Fei H., Thomas L., Thomas G., Tooze S.A. (2003). AP-1 recruitment to VAMP4 is modulated by phosphorylation-dependent binding of PACS-1. EMBO Rep..

[bib0065] Holt M., Cooke A., Wu M.M., Lagnado L. (2003). Bulk membrane retrieval in the synaptic terminal of retinal bipolar cells. J. Neurosci..

[bib0070] Kavalali E.T., Jorgensen E.M. (2014). Visualizing presynaptic function. Nat. Neurosci..

[bib0075] Kelly B.T., Owen D.J. (2011). Endocytic sorting of transmembrane protein cargo. Curr. Opin. Cell Biol..

[bib0080] Kokotos A.C., Cousin M.A. (2015). Synaptic vesicle generation from central nerve terminal endosomes. Traffic.

[bib0085] Kononenko N.L., Puchkov D., Classen G.A., Walter A.M., Pechstein A., Sawade L., Kaempf N., Trimbuch T., Lorenz D., Rosenmund C., Maritzen T., Haucke V. (2014). Clathrin/AP-2 mediate synaptic vesicle reformation from endosome-like vacuoles but are not essential for membrane retrieval at central synapses. Neuron.

[bib0090] Li Z., Burrone J., Tyler W.J., Hartman K.N., Albeanu D.F., Murthy V.N. (2005). Synaptic vesicle recycling studied in transgenic mice expressing synaptopHluorin. Proc. Natl. Acad. Sci. U. S. A..

[bib0095] Linares-Clemente P., Rozas J.L., Mircheski J., Garcia-Junco-Clemente P., Martinez-Lopez J.A., Nieto-Gonzalez J.L., Vazquez M.E., Pintado C.O., Fernandez-Chacon R. (2015). Different dynamin blockers interfere with distinct phases of synaptic endocytosis during stimulation in motoneurones. J. Physiol..

[bib0100] Nathanson J.L., Yanagawa Y., Obata K., Callaway E.M. (2009). Preferential labeling of inhibitory and excitatory cortical neurons by endogenous tropism of adeno-associated virus and lentivirus vectors. Neuroscience.

[bib0105] Nicholson-Fish J.C., Kokotos A.C., Gillingwater T.H., Smillie K.J., Cousin M.A. (2015). VAMP4 is an essential cargo molecule for activity-dependent bulk endocytosis. Neuron.

[bib0110] Peden A.A., Park G.Y., Scheller R.H. (2001). The Di-leucine motif of vesicle-associated membrane protein 4 is required for its localization and AP-1 binding. J. Biol. Chem..

[bib0115] Raingo J., Khvotchev M., Liu P., Darios F., Li Y.C., Ramirez D.M., Adachi M., Lemieux P., Toth K., Davletov B., Kavalali E.T. (2012). VAMP4 directs synaptic vesicles to a pool that selectively maintains asynchronous neurotransmission. Nat. Neurosci..

[bib0120] Ramirez D.M., Khvotchev M., Trauterman B., Kavalali E.T. (2012). Vti1a identifies a vesicle pool that preferentially recycles at rest and maintains spontaneous neurotransmission. Neuron.

[bib0125] Richards D.A., Guatimosim C., Betz W.J. (2000). Two endocytic recycling routes selectively fill two vesicle pools in frog motor nerve terminals. Neuron.

[bib0130] Saheki Y., De Camilli P. (2012). Synaptic vesicle endocytosis. Cold Spring Harb. Perspect. Biol..

[bib0135] Sankaranarayanan S., Ryan T.A. (2000). Real-time measurements of vesicle-SNARE recycling in synapses of the central nervous system. Nat. Cell Biol..

[bib0140] Takamori S. (2006). Molecular anatomy of a trafficking organelle. Cell.

[bib0145] Watanabe S., Rost B.R., Camacho-Perez M., Davis M.W., Sohl-Kielczynski B., Rosenmund C., Jorgensen E.M. (2013). Ultrafast endocytosis at mouse hippocampal synapses. Nature.

[bib0150] Watanabe S., Trimbuch T., Camacho-Perez M., Rost B.R., Brokowski B., Sohl-Kielczynski B., Felies A., Davis M.W., Rosenmund C., Jorgensen E.M. (2014). Clathrin regenerates synaptic vesicles from endosomes. Nature.

[bib0155] Wenzel E.M., Morton A., Ebert K., Welzel O., Kornhuber J., Cousin M.A., Groemer T.W. (2012). Key physiological parameters dictate triggering of activity-dependent bulk endocytosis in hippocampal synapses. PLoS One.

[bib0160] Wilhelm B.G., Mandad S., Truckenbrodt S., Krohnert K., Schafer C., Rammner B., Koo S.J., Classen G.A., Krauss M., Haucke V., Urlaub H., Rizzoli S.O. (2014). Composition of isolated synaptic boutons reveals the amounts of vesicle trafficking proteins. Science.

[bib0165] Wu W., Wu L.G. (2007). Rapid bulk endocytosis and its kinetics of fission pore closure at a central synapse. Proc. Natl. Acad. Sci. U. S. A..

[bib0170] Zhu Y., Xu J., Heinemann S.F. (2009). Two pathways of synaptic vesicle retrieval revealed by single-vesicle imaging. Neuron.

